# αvβ6- and αvβ8-Integrins Serve As Interchangeable Receptors for HSV gH/gL to Promote Endocytosis and Activation of Membrane Fusion

**DOI:** 10.1371/journal.ppat.1003806

**Published:** 2013-12-19

**Authors:** Tatiana Gianni, Stefano Salvioli, Liudmila S. Chesnokova, Lindsey M. Hutt-Fletcher, Gabriella Campadelli-Fiume

**Affiliations:** 1 Department of Experimental, Diagnostic and Specialty Medicine, Alma Mater Studiorum–University of Bologna, Bologna, Italy; 2 Department of Microbiology and Immunology, Center for Molecular and Tumor Virology and Feist-Weiller Cancer Center, Louisiana State University Health Sciences Center, Shreveport, Louisiana, United States of America; University of California, Irvine, United States of America

## Abstract

Herpes simplex virus (HSV) - and herpesviruses in general - encode for a multipartite entry/fusion apparatus. In HSV it consists of the HSV-specific glycoprotein D (gD), and three additional glycoproteins, gH/gL and gB, conserved across the *Herpesviridae* family and responsible for the execution of fusion. According to the current model, upon receptor binding, gD propagates the activation to gH/gL and to gB in a cascade fashion. Questions remain about how the cascade of activation is controlled and how it is synchronized with virion endocytosis, to avoid premature activation and exhaustion of the glycoproteins. We considered the possibility that such control might be carried out by as yet unknown receptors. Indeed, receptors for HSV gB, but not for gH/gL, have been described. In other members of the *Herpesviridae* family, such as Epstein-Barr virus, integrin receptors bind gH/gL and trigger conformational changes in the glycoproteins. We report that αvβ6- and αvβ8-integrins serve as receptors for HSV entry into experimental models of keratinocytes and other epithelial and neuronal cells. Evidence rests on loss of function experiments, in which integrins were blocked by antibodies or silenced, and gain of function experiments in which αvβ6-integrin was expressed in integrin-negative cells. αvβ6- and αvβ8-integrins acted independently and are thus interchangeable. Both bind gH/gL with high affinity. The interaction profoundly affects the route of HSV entry and directs the virus to acidic endosomes. In the case of αvβ8, but not αvβ6-integrin, the portal of entry is located at lipid microdomains and requires dynamin 2. Thus, a major role of αvβ6- or αvβ8-integrin in HSV infection appears to be to function as gH/gL receptors and to promote virus endocytosis. We propose that placing the gH/gL activation under the integrin trigger point enables HSV to synchronize virion endocytosis with the cascade of glycoprotein activation that culminates in execution of fusion.

## Author Summary

In order to infect their hosts and cause disease, viruses must enter their host cells. The human pathogen herpes simplex virus (HSV) - and herpesviruses in general - are equipped with a complex, multipartite entry apparatus, made of four glycoproteins – gD, gH/gL, gB. These glycoproteins must be activated in a timely, coordinated manner. According to the current model, the flux of activation goes from receptor-bound gD, to gH/gL and gB. The premature activation, and hence exhaustion of the glycoproteins must also be prevented. We report on a checkpoint at the gH/gL level. Specifically, αvβ6- and αvβ8-integrins serve as receptors for HSV entry into keratinocytes and other epithelial and neuronal cells. Both bind gH/gL with high affinity. The interaction profoundly affects the pathway of HSV entry, promoting HSV endocytosis into acidic endosomes. For αvβ8-integrin, the portal of entry is at lipid microdomains and requires dynamin 2. We propose that, by placing the activation of gH/gL under control of an integrin trigger point, HSV can synchronize virion endocytosis with the cascade of activation that culminates in the execution of fusion between the virion envelope and cellular membranes.

## Introduction

The glycoproteins of enveloped virions fulfill three major functions to enable virus entry into target cells; the attachment of virions to cells, a step that partly determines the type of cells that the virus targets, hence the viral tropism; the triggering of fusion, i.e. the activation of the fusion machinery, and the execution of fusion. For a number of viruses, a fourth event occurs between these steps, virion internalization by endocytosis, or macropinocytosis. The domains responsible for all these activities are often localized in one or two glycoproteins; this is the case for example for ortho-, paramyxo- and retroviruses. Virion glycoproteins can be considered ready-to-use machines that need to undergo a transition in conformation from the metastable fusion-inactive to the fusion-active form, in order to induce the merging of the two membranes - that of the virion and that of cell - so that lipids are mixed and fusion is executed [1]. A fundamental aspect of the process is that the steps are sequentially ordered and coordinated, to ensure that the glycoprotein transition takes place only after the virus has attached to the cells. Indeed, a premature activation would irreversibly exhaust the fusogenic potential of the virion glycoproteins, and lead to failure to infect. A key question is therefore how the timing of glycoprotein transition and activation is controlled. Essentially, there are two strategies. Either the glycoprotein transition is dependent on the glycoprotein encounter with the cognate cellular receptor, or on the low pH of the endosomal compartment. These levels of control guarantee that the virion fusion machinery is only active after the virus has attached to cells, or, for those viruses which undergo internalization, after they have been endocytosed and the endosomal pH has been lowered. According to this view, two major functions of cellular receptors are determination of viral tropism and triggering of fusion.

Herpes simplex virus (HSV), and herpesviruses in general, exhibit a high level of complexity since they encode a multipartite entry/fusion machinery [2], [3]. Some of the herpesvirus glycoproteins are species-specific; they play a role in the initial steps of virus entry and interact with receptors that belong to variety of molecular families. A prototypic example is HSV glycoprotein D (gD) that binds alternatively two major receptors, nectin1 and herpesvirus entry mediator (HVEM) [4]–[6]. It functions as a determinant of HSV tropism and as the trigger of fusion capable to activate the downstream glycoproteins gH/gL and gB [7]–[10]. Conformational modification of gD following receptor binding was inferred by structural, biochemical and molecular biology approaches [7], [8], [11]–[13]. gD encodes a specific domain – the profusion domain - that interacts with, or at least propagates the activation to gH/gL, and thereafter or simultaneously activates gB in a cascade fashion [9], [10], [14], [15]. Species-specific glycoproteins among human herpesiviruses include Epstein Barr virus (EBV) gp42, which binds major histocompatibility complex II to enable virus entry into lymphocytic cells [16], [17]; the human cytomegalovirus (HCMV) glycoproteins of the 128–131 locus [18], which confer endothelial and epithelial tropism; the human herpesvirus 6 (HHV-6) gQ1-gQ2 that enable gH/gL interaction with CD134 [19].

In addition to the species-specific glycoproteins, three glycoproteins - the heterodimer gH/gL and gB - constitute the conserved core fusion apparatus across the *Herpesviridae* family. gB has structural features typical of fusion glycoproteins. In contrast, gH/gL does not resemble any known structure. A number of approaches support the view that gH/gL acts as an intermediate in the activation cascade of HSV entry/fusion glycoproteins. Remarkably, for some herpesviruses, gH/gL or gB serve as major receptor-binding glycoproteins and therefore their activation may be under the direct control of encounter with the receptor. Thus, members of the integrin family serve as receptors for EBV and HCMV gH/gL [20]–[22], and for HHV-8 gB [23]. EphrinA2 receptor serves as a receptor for HHV-8 gH/gL [24], [25]. Receptors for HSV gB and varicella zoster virus (VZV) gB include paired immunoglobulin-like type 2 receptor alpha (PILRα), myelin associated glycoprotein, and non-muscle myosin IIA [26]–[28]; their role in virus entry remains to be fully elucidated.

We have recently discovered two additional functions of cellular receptors for HSV, namely that they serve as routing factors to define the pathway of entry and that they serve as viral sensors capable of initiating the innate response of the cell, and thus coupling virus entry to the innate response. Specifically, HSV enters different cells by different pathways. αvβ3-integrin is a determinant in the choice of the HSV pathway of entry which routes the HSV receptor nectin1, and consequently HSV, to lipid microdomains [29], [30]. In this way, αvβ3-integrin enables entry of HSV through a pathway dependent on lipid microdomains, dynamin 2 and acidic endosomes. αvβ3-integrin interacts at low affinity with HSV gH/gL [31], [32]. Furthermore, αvβ3-integrin also binds Toll-like receptor 2 (TLR2), which, in turn, binds gH/gL[33]. In this way αvβ3-integrin senses HSV and cooperates with and reinforces the TLR2–dependent response [31]. A signaling cascade is initiated that leads to activation of the transcription factor NF-κB, and to production of intereferon (IFN) α and β, the major innate defenses of the cell against HSV [31].

Here, we investigated whether integrins other than αvβ3 play a role in HSV entry. We investigated the αv group of integrins, which are preferentially expressed in epithelial cells, and bind their ligands through the RGD domain. αvβ6-integrin is upregulated in epithelial malignancies. We report that αvβ6- and αvβ8-integrins serve as interchangeable HSV receptors for entry into keratinocytes, other epithelial and neuronal cells. Each integrin acted independently of the other. Both bind gH/gL at high affinity. This interaction profoundly affects the pathway of HSV entry, which takes the way of acidic endosomes. In the case of αvβ8-integrin, the portal of entry is located at lipid microdomains and requires dynamin 2. In the case of αvβ6-integrin, the portal of entry is located outside the lipid microdomains and does not need dynamin 2. We propose that the major role of αvβ6- and αvβ8-integrins as gH/gL receptors is to promote HSV endocytosis, and thereby to synchronize virion internalization with the cascade of glycoprotein activation.

## Results

### A soluble form of HSV gH/gL binds αvβ6- and αvβ8-integrins at high affinity

**Figure 1:**
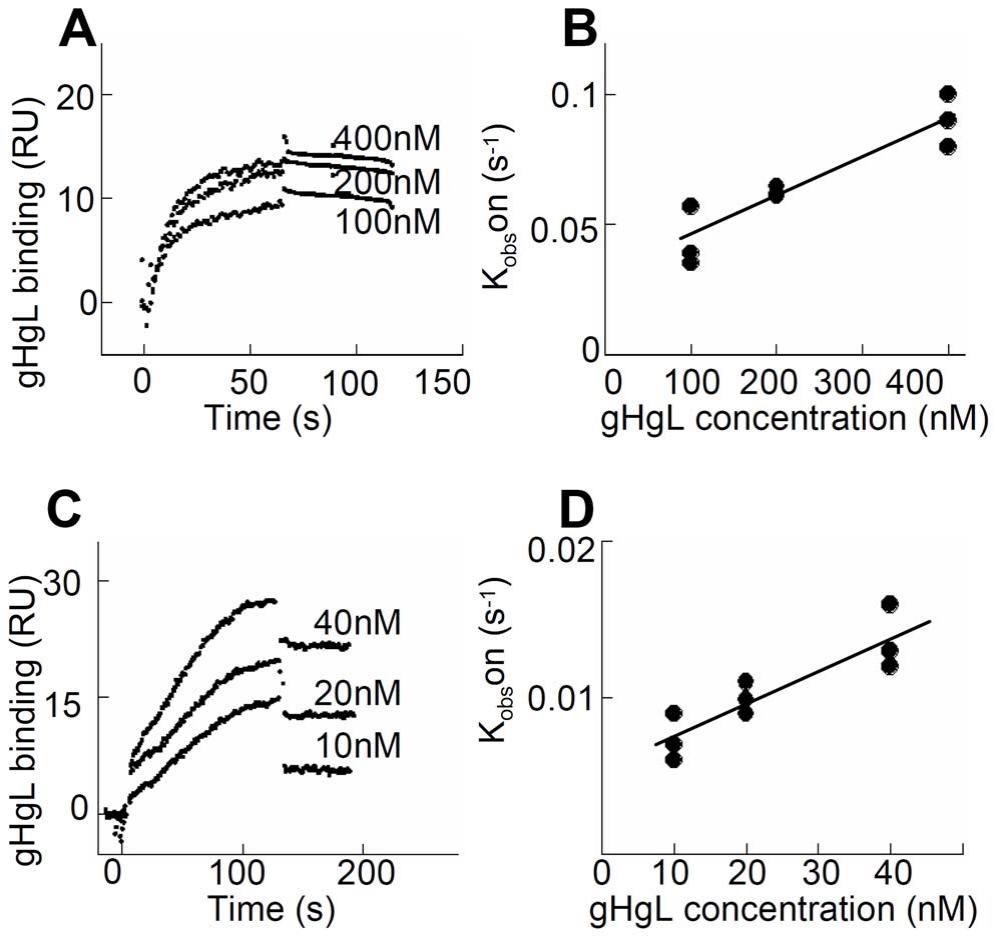
A soluble form of HSV gH/gL binds soluble αvβ6- and αvβ8–integrin at high affinity. (A and C) Surface Plasmon Resonsance spectroscopy was performed to evaluate binding of different concentrations of gH/gL, as indicated, to immobilized αvβ6-integrin (A) and αvβ8-integrin (C). (B and D) Linear plots of *k*_obs_ versus gH/gL concentration for the gH/gL interaction with αvβ6-integrin (B) and αvβ8-integrin (D). The intersection points with the Y axes correspond to the dissociation rates constants k_off_ and the slopes to the association rate constants k_on_. Three independent experiments were performed for each integrin and results were plotted together without normalizing.

The interaction between HSV gH/gL and integrins was explored using surface plasmon resonance spectroscopy of soluble integrins and soluble gH/gL. A kinetic analysis of integrin binding to increasing concentrations of soluble gH/gL indicated that gH/gL interacted with both αvβ6-integrin and with αvβ8-integrin (Fig. 1). To obtain rate constants of gH/gL binding and to calculate a dissociation constant (K_D_), k_obs_ was plotted against the concentration of gH/gL. The dependence of k_obs_ on gH/gL concentration was described by a linear function suggesting that the interaction has a single step. The intersection point with the Y-axes corresponds to a dissociation rate constant k_off_, and the slope to an associate rate constant k_on_. The rate constants and the resulting K_D_ values (Table 1) are indicative of a high affinity interaction. In contrast, no interaction between integrin αvβ5 and gH/gL was detected.

**Table 1:** Kinetic constants (±SD) for gH/gL binding to soluble truncated integrins.

Integrin	k_on_ (M^−1^s^−1^)	k_off_(s^−1^)	K_D_(M)
αvβ6	152,000±2,300	0.0018±0.0004	(1.2±0.4)×10^−8^
αvβ8	207,000±12,000	0.005±0.001	(2.7±0.7)×10^−8^
αvβ5	no binding	-	-

### Expression of αvβ6- and αvβ8-integrins in epithelial cell lines and silencing by siRNAs

**Figure 2:**
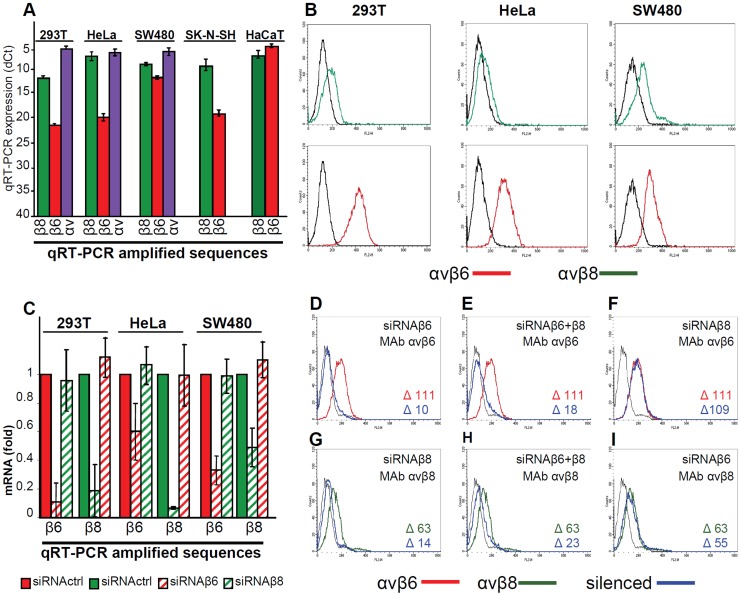
Expression of αvβ6- and αvβ8-integrins in epithelial 293T, HeLa, SW480, SK-N-SH and HaCaT cell lines and silencing by siRNA. (A) q-RT-PCR quantification of αv- (blue), β6- (red) and β8- (green) integrin subunits in 293T, HeLa, SW480, SK-N-SH and HaCaT cells. Results are expressed as dCt, relative to GAPDH (Glyceraldehyde 3-phosphate dehydrogenase). Of note, the lower the dCt value, the higher the extent of expression. (B) Expression of αvβ6- (red) and αvβ8 (green) -integrin as detected by flow cytometry. The indicated cells were reacted with MAb to αvβ6 or αvβ8-integrins. Abscissa, fluorescence intensity. (C–I) Relative change in β6- and β8-integrin expression between control mock-silenced cells (siRNActrl) and cells silenced for β6- or β8–integrins (named siRNAβ6 and siRNAβ8 respectively) (C) Expression was determined by q-RT-PCR by means of the 2^−ΔΔCt^ method. The fold expression in siRNActrl cells was made equal to 1. (D–I) Flow cytometry determination of αvβ6 (red) and αvβ8 (green) –integrin expression in mock-silenced (siRNActrl), or silenced for β6- (D, I), β8- (F, G), or β6-plus β8- (E, H) integrins 293T cells. The integrin-silenced cells are in blue. The figures within each panel refer to difference (Δ) in fluorescence intensity of the stained cells and unspecific fluorescence (black line).

αvβ6-integrin is expressed in epithelial cells and upregulated in epithelial cancer cells. αvβ8-integrin is expressed in some types of epithelial cells, as well as in glial and dendritic cells [34], [35]. We determined the expression of αvβ6- and of αvβ8-integrins in 293T cells (epithelial cells transformed by an adenovirus fragment), HeLa, colon carcinoma SW480, keratinocyte HaCaT and in the neuronal SK-N-SH cell lines. By qRT-PCR (Fig. 2A) β8-integrin was expressed at higher levels than β6-integrin in 293T, HeLa and SK-N-SH cells, and to comparably high levels in SW480 and HaCaT cells. The epithelial cell lines were analyzed also by flow cytometry; they exhibited the highest fluorescence intensity with the antibody to αvβ6-integrin (Fig. 2 B). A direct comparison between αvβ6- and αvβ8-integrin extent of expression in a same cell line can not be performed by flow cytometry, since reactivity in this assay is strongly influenced by the properties of the antibody being used.

The extent of silencing achieved with specific si-RNAs to β6- or to β8-integrin as measured by q-RT-PCR is shown in Fig. 2 C. The extent of silencing was greater than 80% for both integrins in 293T cells. It was around 75 and 50% for β6- and β8-integrin in SW480 cells, and about 40% and 90% for β6- and β8-integrin in HeLa cells. Silencing was specific as there was no off-target effect in any of the cell line. Thus, when β6-integrin was silenced, β8-integrin mRNA level was not decreased. Similarly, when β8-integrin was silenced, β6-integrin mRNA level was not decreased. Silencing was confirmed by flow cytometry analysis in 293T cells and expressed as median fluorescence intensity for β6-integrin (Fig. 2 D–F), and for β8-integrin (Fig. 2 G–I). The lack of off-target was confirmed by flow cytometry. (Fig. 2 F, I). The effect of the double silencing on each of the two integrins could not be differentiated from that of single silencing (Fig. 2 E and H).

### MAbs to αvβ6- or αvβ8-integrin or silencing of β6- or β8-integrin inhibit HSV-1 infection

**Figure 3:**
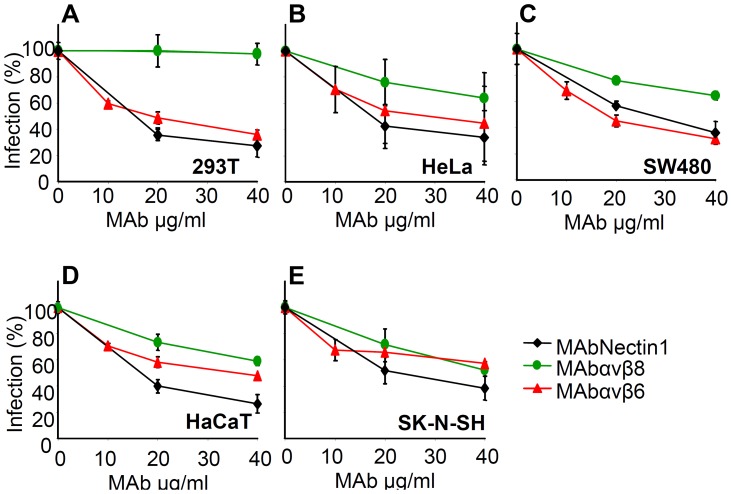
Inhibition of HSV-1 infection by function blocking MAbs to αvβ6- or αvβ8–integrin. 293T (A), HeLa (B), SW480 (C), HaCaT (D), SK-N-SH (E) cells were exposed to increasing amounts of MAb to nectin1 (black diamond), to αvβ6-integrin (red triangle), to αvβ8- integrin (green circle) for 1 h, infected with R8102 (3 pfu/cell) in the same medium, and overlaid with MAb-containing mediun until harvest at 6–8 h after infection. The extent of infection was quantified from Lac-Z gene engineered in the viral genome under the immediate-early α27-promoter. Extent of β-Gal activity, measured as conversion of ONPG substrate, reflects the amount of infection. Each point represents the average of triplicates. ONPG conversion was measured by O.D. reading at 405 nm [5]. 100% infection is the value obtained with murin IgG used as a control. Bars show SD.

To define if αvβ6- and αvβ8-integrin play a role in HSV infection, we tested the effect of function-blocking monoclonal antibodies (MAbs) to αvβ6-integrin (MAb 2077Z), or αvβ8-integrin (MAb 37E1), on HSV-1 infection. The neutralizing R1.302 MAb to nectin1 was used as a positive control. Cells were preincubated with increasing amounts of MAbs, and infected in the presence of MAbs with the recombinant R8102 which carries a Lac-Z reporter gene. A large body of evidence indicates that the extent of β-galactosidase (β-gal) expression directly reflects the extent of infection [4], [5]. Fig. 3A–E shows that MAbs to αvβ6 or to αvβ8-integrin inhibited R8102 infection in a dose-dependent manner in all cells. The only exception was MAb to αvβ8-integrin which failed to inhibit infection in 293T cells, even though these cells exhibited cell surface expression of this integrin. Thus, both αvβ6 and αvβ8-integrins play a critical role in HSV infection of epithelial, keratynocytic and neuronal cells.

**Figure 4:**
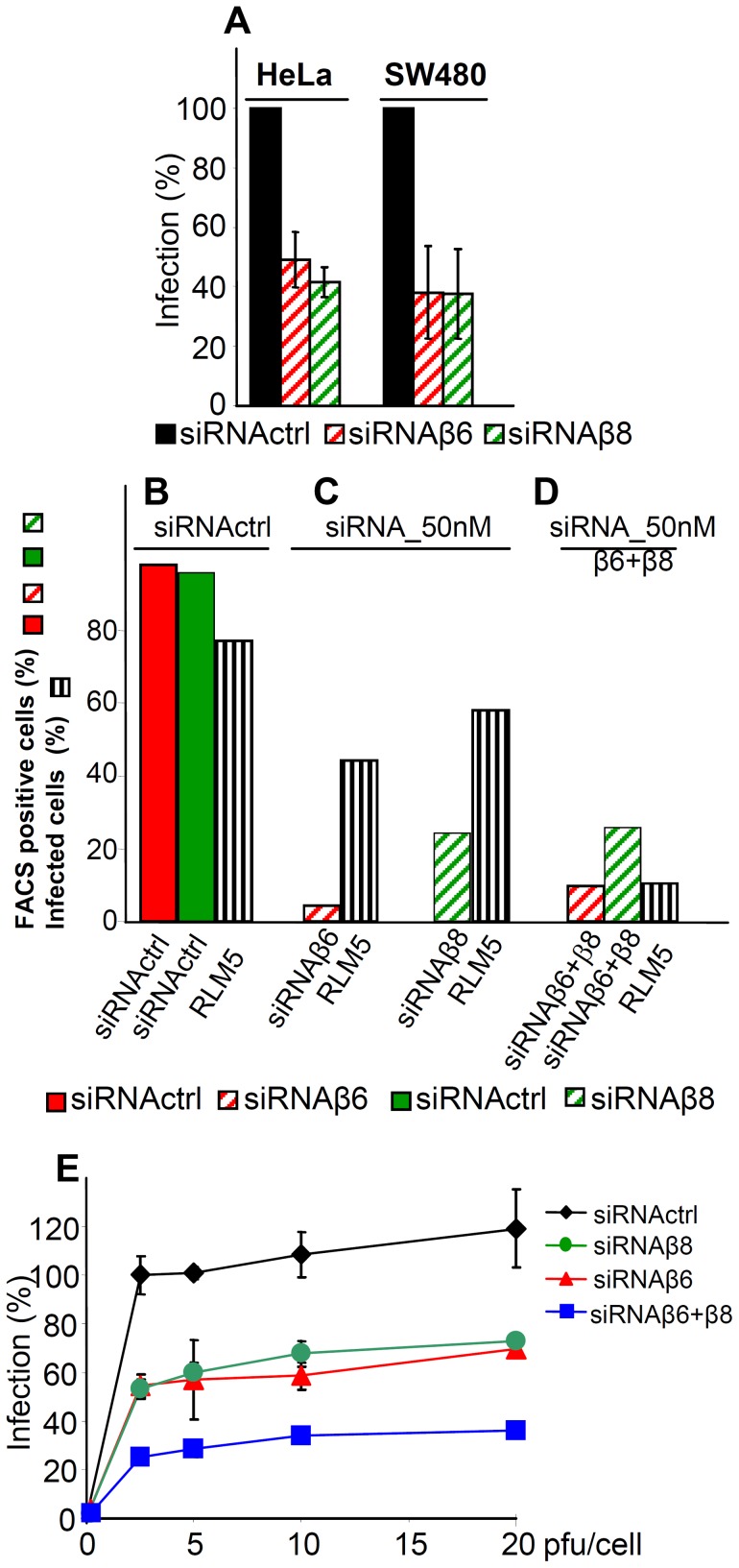
Inhibition of HSV-1 infection by silencing of β6- or β8-integrin. (A) Extent of R8102 infection in silenced cells was quantified as detailed legend to Fig. 3. 100% infection is the value obtained with siRNActrl cells. Values represent the average of quadruplicates. Bars show SD. (B–D) Red or green columns represent extent of β6 or of β8-integrin expression in 293 T cells, either mock-silenced (full columns), or silenced (hatched columns). Striped black columns represent extent infection with RLM5 as detected by FACS. (E) Inhibition of R8102 infection in 293T cells silenced with siRNActrl (black diamond), or with siRNA to β6- (red triangle), β8- (green circle), or β6-plus β8- (blue square) integrins. Cells were infected at increasing MOI (pfu/cell), and harvested at 16 h after infection, as detailed in the legend to Fig. 3. each point represents the average of triplicates ± SD.

As a second approach, silencing of β6- or of β8-integrin with 50 nM si-RNA inhibited infection in the three lines - HeLa and SW480 (Fig. 4A) and 293T (Fig. 4 B–E). Thus, two assays concordantly indicate a requirement for αvβ6- and αvβ8-integrins in HSV infection.

Because all cell lines tested contained both αvβ6-integrin and αvβ8-integrin, we asked whether each one of the two integrins is required independently one of the other, or whether they play an interchangeable role. In the latter case, the expectation is that in cells silenced for a single integrin, the extent of inhibition of infection is smaller than the extent of silencing. By contrast, in cells simultaneously silenced for both integrins, the inhibition of infection is expected to be higher than that achieved in cells silenced for a single integrin. Fig. 4B–D shows the extent of silencing as percentage of positive cells; their mean fluorescence intensities is shown in Fig. 2 E, H. Indeed, in 293T cells silenced for either or the other integrin, infection was reduced to a lower extent than expression. When the two integrins were simultaneously silenced, infection dropped to about 10%. In a replicate experiment, R8102 infection in singly or doubly silenced cells was quantified by means of β-gal. Fig. 4 E shows that infection was inhibited by about 75% in double-silenced cells, and by about 50% in singly silenced cells. The results indicate that αvβ6-integrin and αvβ8-integrin play each a critical role in HSV infection, each independently of the other. Thus, they appear to act in an interchangeable fashion.

### Gain of function: Expression of αvβ6-integrin in integrin-negative K562 cells increases HSV infection

**Figure 5:**
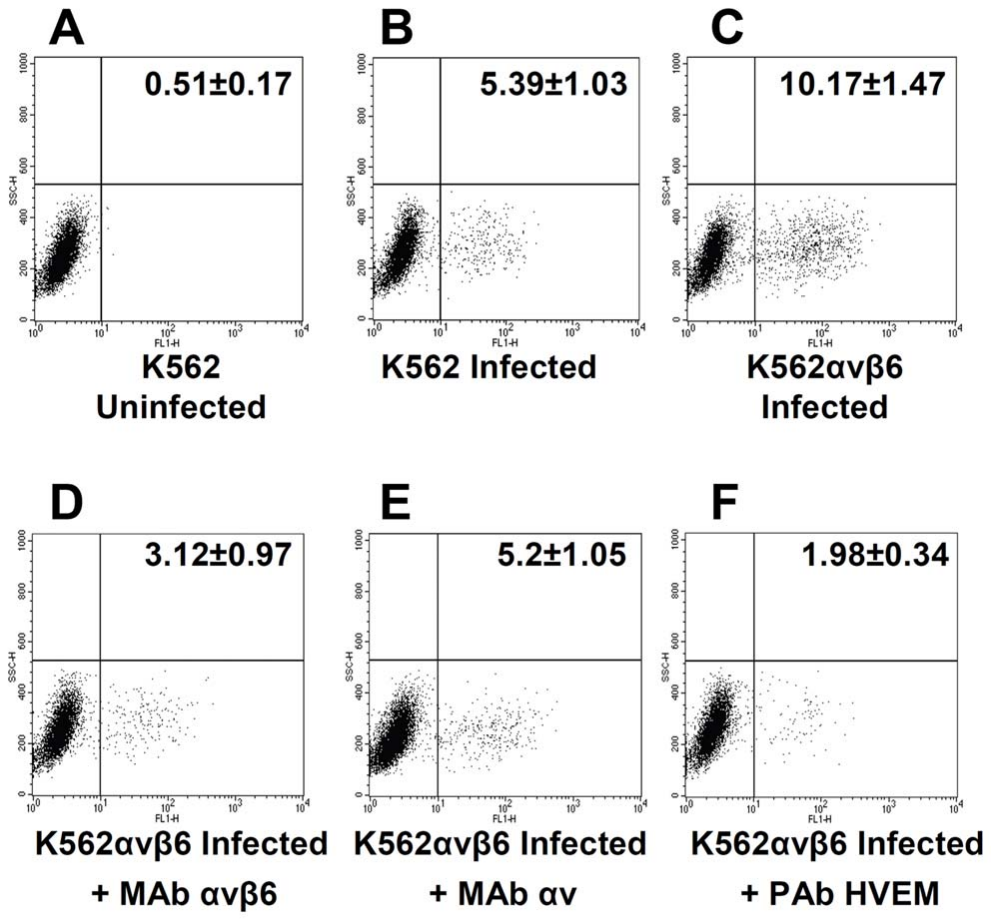
Gain of function experiment shows increase in infection with RLM5 in K562 cells following expression of αvβ6-integrin. (A, B) wt-K562 cells were uninfected (A) or infected with RLM5 (B). (C–F) K562 cells expressing αvβ6-integrin (K562_αvβ6_) cells were infected with RLM5. (D–F) Inhibition of RLM5 infection in K562_αvβ6_ cells exposed to MAb to αvβ6-integrin (D), or to αv-integrin (E) or to PAb to HVEM (F) for 1 h prior to infection with RLM5 in the same medium, and incubated with MAb-containing medium until harvest at 6 h after infection. The extent of infection was determined as percentage of cells expressing GFP. The figures reported in each panel represent percentage of cells in the lower right quadrant and represent the mean and standard deviation of four independent experiments.

To confirm the role of αvβ6-integrin in HSV infection, we performed a gain of function experiment. The myelocytic cell line K562 expresses a very limited number of integrins, predominantly α5β1, and expresses no detectable αv [36]. A stable cell line in which about 20% of cells transgenically express αvβ6-integrin (K562_αvβ6_) was obtained from Dr S. Blystone. The wt-K562 and K562_αvβ6_ cells were infected with RLM5 HSV mutant, which carries the GFP moiety and enables flow cytometry quantification of infection. To authenticate the results, infection was carried in the presence or absence of MAb 2077Z to αvβ6-integrin, MAb L230 to αv-subunit, or polyclonal antibody (PAb) R140 to HVEM as a control. Myelocytic cells, including K562 cells, are hardly infected by HSV and likely restrict infection at entry and post-entry steps [37]. Fig. 5 shows a representative experiment, and reports figures relative to the average percentage infected cells, as determined in four independent experiments. It can be seen that the number of infected cells doubled in αvβ6-integrin^+^ K562 cells, relative to wt-K562 cells (10.17 *versus* 5.39%). Extent of infection in αvβ6-integrin^+^ K562 cells was strongly inhibited when infection took place in the presence of MAb 2077Z to αvβ6-integrin (3.12%), or MAb L230 to αv-subunit (5.2%), or in the presence of anti-HVEM PAb R140 (1.98%). Although the percentage of infected cells is rather low, in agreement with well-known resistance to HSV infection exhibited by this type of cells [37], the results clearly indicate that expression of αvβ6-integrin enhances HSV infection of K562 cells; furthermore, the single αvβ6-integrin – in the absence of αvβ8-integrin - was sufficient to induce the increase in infection. The latter finding reinforces the conclusion drawn from the previous series of experiments that each of the two integrins is sufficient to promote HSV infection.

### Silencing of β6- or β8-integrin reduces HSV-1 attachment to cells

**Figure 6:**
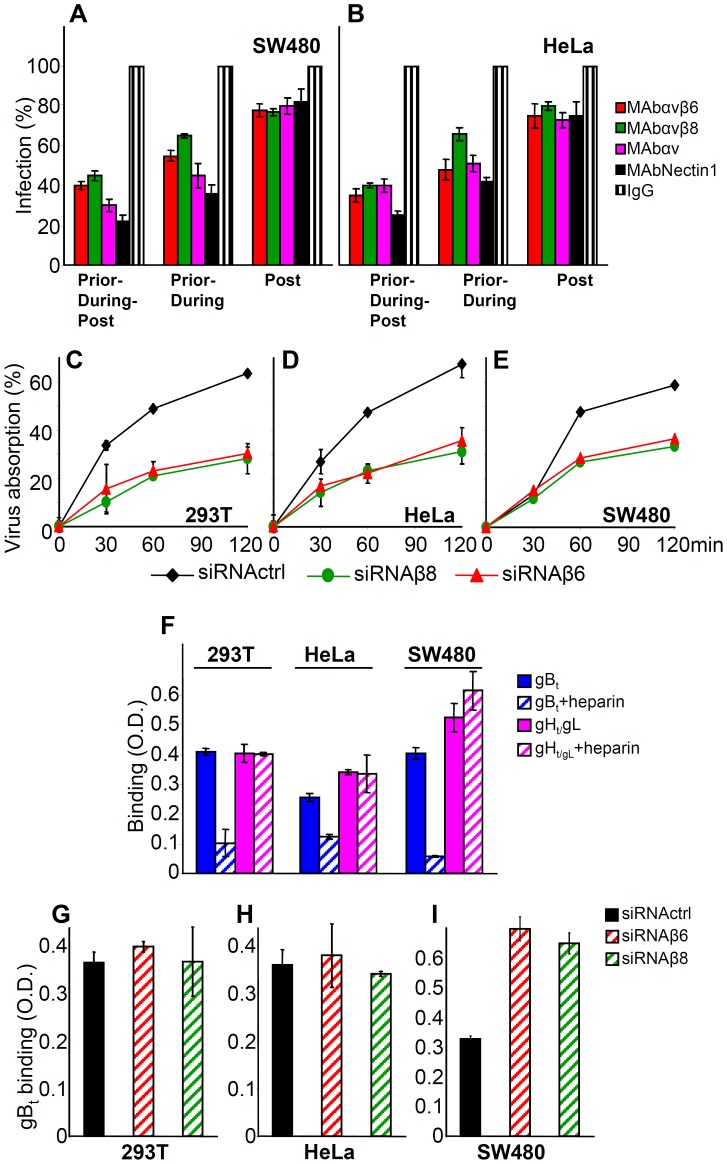
Steps in HSV infection inhibited by function-blocking MAbs to αvβ6- and αvβ8–integrins, or by integrin silencing. (A, B) The indicated cells were exposed to MAb 2077Z to αvβ6-integrin, MAb 37E1 to αvβ8–integrin, MAb L230 to αv–integrin, MAb R1.302 to nectin1, or control IgGs. In the “Prior-during-post” treatment cells were exposed to the antibodies from 1 h prior to infection till time of harvest, at 6–8 h after infection. In the “Prior-during” treatment cells were exposed to the antibodies from 1 h prior to infection and during virus absorption. In the “Post” treatment, cells were exposed to the antibodies from the end of virus absorption till harvesting. Cells were infected with R8102 (3 pfu/cell). Infection was quantified in triplicates as detailed in the legend to Fig. 3, and expressed as percentage relative to cells treated with control IgGs. (C–E) Inhibition of R8102 absorption to integrin-silenced cells. β6- or β8–integrins were silenced by siRNAs. Control cells received siRNA control. Cells silenced for 2–3 days were infected with R8102 for 120 min at 4°C. Aliquots of the viral inoculum were withdrawn in duplicates at the indicated times, and immediately titrated in Vero cells. Extent of virus absorption is expressed as percentage of the amount of virus present in the inoculum at 0 time. Bars show SD. (F) The binding of gB_t_, and not that of gH_t_/gL, to cells is inhibited by heparin. One-StrEP tagged gB_t_, gH_t_/gL or GFP_t_ (2 µM each), the latter as a negative control, were preincubated or not with heparin (5 µg/ml) for 1 h at 4°C, and then added to cells grown in 96-well plates, for 1 h at 4°C. Binding was detected by means of HRP-conjugated MAb to the One-StrEP tag and the *o*-phenylenediamine substrate [41]. Each point represents the average of triplicates. The values obtained with GFP_t_ were considered as background values and subtracted. Bars represent SD. (G–I). Determination of heparin sulphate binding sites by means of gB_t_ to integrin silenced and control-silenced cells. (G–I). 293T (G), HeLa (H) and SW480 (I) cells, control-silenced or integrn-silenced, were exposed to gB_t_, pretreated or not heparin. Binding was detect as descibed in panel F. Columns represent the binding of gB_t_, after subtraction of the values obtained in the presence of heparin. Each column represents the average of triplicates. Bars denote SD.

The next series of experiments was designed to shed light on the role played by αvβ6- or αvβ8-integrin in HSV infection. First, we defined the step(s) in the HSV entry process in which they participate. Cells were exposed to anti-integrin antibodies prior, during and post virus absorption to cells, prior and during virus absorption, or only after virus absorption to cells. The extent of inhibition of infection was then quantified. These experiments could not be performed in 293T cells, as in these cells the anti- αvβ8-integrin MAb fails to inhibit infection (see, Fig. 3A). As shown in Fig. 6 A and B, the highest extent of inhibition was seen when MAbs were present prior, -during and post virus absorption to cells. The decrease seen when Abs were present prior and during, but not post virus absorption was somewhat lower. The presence of Abs after virus absorption decreased infection by about 20%. The results are consistent with the view that blocking αvβ6 or αvβ8-integrins with antibodies inhibits virus infection at different steps in the virus entry process, and suggest a role for integrins at attachment and post-attachment steps.

To verify a role for αvβ6- and αvβ8-integrin in HSV attachment, we measured the effect of integrin silencing on HSV absorption to cells at 4°C. Virus absorption was quantified as a decrease in infectious virions present in the inoculum. Fig. 6 C–E shows that virus absorption was inhibited by about 50% in β6- or β8-integrin silenced 293T, HeLa, and SW480 cells, indicating that αvβ6- and αvβ8-integrins contribute to virus absorption to cells. This contribution is small relative to that exerted by heparan sulphate. In cells where heparan sulphate is removed by treatment with heparitinase, in mutants defective in heparan sulphate biosynthesis, or in cells in which infection is competitively blocked by heparin, the decrease in infection is about hundred fold [38] [39]. Notwithstanding this consideration, we asked whether the decrease in HSV absorption seen in cells silenced for β6- or β8-integrins was to be attributed to the decrease in integrins, or reflected an indirect effect of integrin silencing on the extent of cell surface expression of heparan sulphate (HS). We exploited the ability of HSV gB to bind HS, and made use of a soluble form of gB (gBt) to quantify the HS binding sites present on the surface of non-silenced *versus* silenced cells. The properties of gB_t_, truncated at aa 730 and tagged with a strep tag (previously named gB_730t-st_) were described [33]. Binding to cell surface was quantified by cell enzyme linked immunosorbent assay (CELISA). Preliminarily, we ascertained that the binding of gB_t_ was competitively inhibited by heparin; in contrast, attachment of a soluble form of gH/gL (gHt/gL) was not inhibited, as expected (Fig. 6 F) [33]. Fig. 6 G – I shows that there was no significant decrease in gB_t_ binding, following β6- or β8-integrin silencing. These results rule out that the decrease in HSV absorption to β6- or β8-integrin-silenced cells (Fig. 6 C–E) was due to a silencing-mediated decrease in HS binding sites.

### αvβ6- and αvβ8-integrins do not substitute for nectin1, yet they greatly increase extent of infection in cells that express nectin1 at low levels

**Figure 7:**
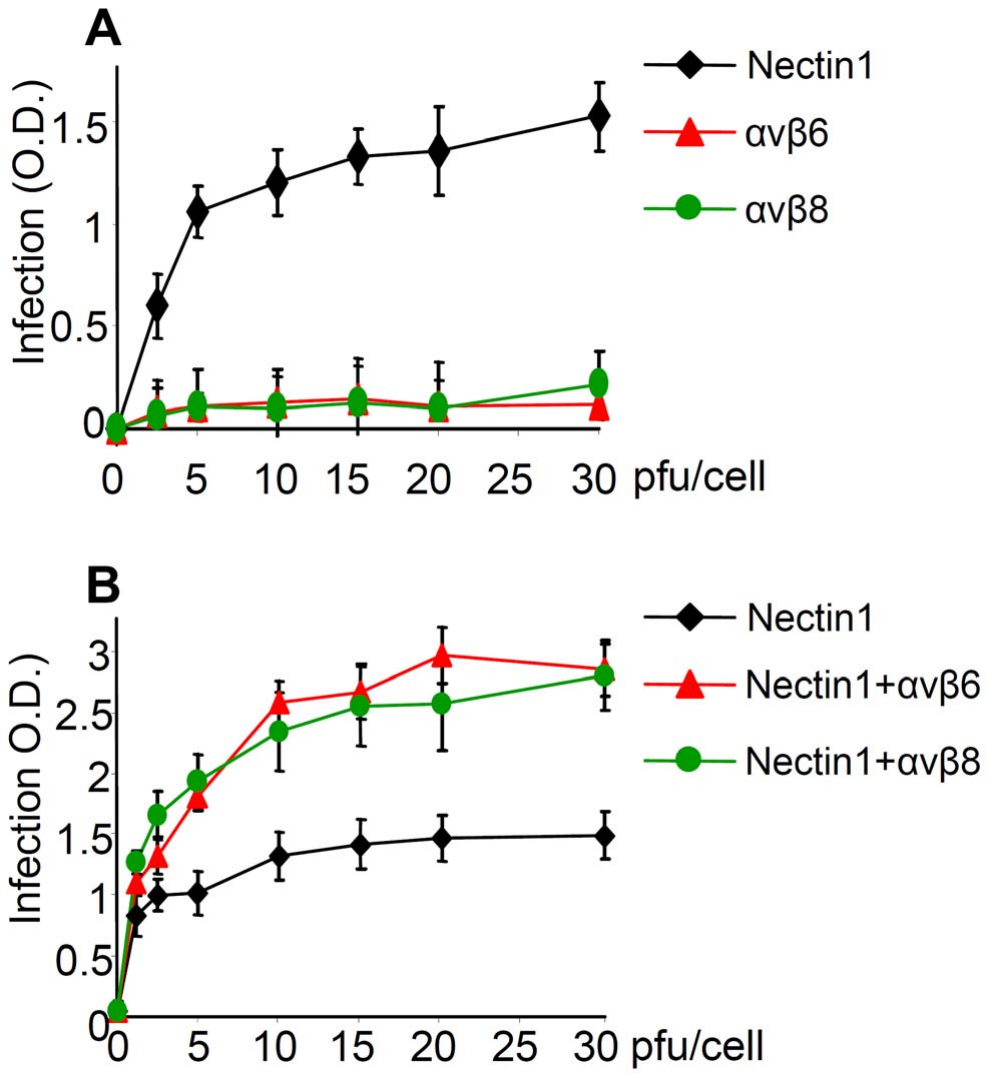
αvβ6 and αvβ8 -integrins do not substitute for nectin 1, yet they increase infection of R8102 in J cells expressing nectin 1 at low level. (A) The receptor-negative J cells were transfected with nectin1 alone, αvβ6-integrin alone, or αvβ8-integrin alone. (B) J cells were transfected with low amount (75 ng DNA/24 well) of nectin1 alone, or with the same amount of nectin1 plus αvβ6-integrin (300 ng DNA/24 well), or plus αvβ8–integrin (300 ng DNA/24 well). 48 h after transfection, cells were infected with R8102, at increasing MOI (2.5–30 pfu/cell) and harvested at 16–18 h after infection. Experimental details as in Fig. 3. Extent of β-Gal activity, measured as ONPG conversion was measured by O.D. reading at 405 nm. Each point represents the average of triplicates. Bars show SD.

For some herpesviruses, e.g. EBV, integrins suffice as receptors for infection of epithelial cells. Here, we asked whether either αvβ6- or αvβ8- integrin suffices as an HSV receptor for entry, or whether their roles are in addition to that of the gD receptors. We made use of the J cells, which are negative for both gD receptors, hence cannot be infected by HSV unless a receptor is transgenically expressed [5]. J cells express endogenous hamster integrins, likely at low levels, hence they are not suitable to test the effect of integrin silencing, or of antibodies to integrins, since the siRNAs and the antibodies were directed to the human orthologs. They were transfected with plasmids encoding for nectin1, or αvβ6-integrin, or αvβ8-integrin (300 ng DNA/well for each plasmid), and then infected with increasing amounts of R8102. Fig. 7 A shows that J cells expressing αvβ6-integrin alone or αvβ8-integrin alone, in the absence of nectin1, did not enable HSV infection, in contrast to cells expressing nectin1 alone. Thus, neither of the two integrins suffices as HSV receptor in cells negative for gD receptors. Parenthetically, in this assay, in the absence of gD receptors, the candidate gB receptor PILR α [40] did not enable infection.

Next, J cells were transfected with low amounts of nectin1 plasmid, plus αvβ6-integrin- or αvβ8-integrin plasmids (75 ng DNA/well for nectin1 plasmid, 300 ng DNA/well each for α and for β integrin-subunits). It can be seen from Fig. 7 B that αvβ6- or αvβ8-integrin each doubled the efficiency of infection attained with nectin1 alone. Thus, αvβ6- and αvβ8-integrin are not sufficient to substitute for nectin1, but they do increase nectin1-dependent infection.

### Infection and cell-to-cell fusion mediated by αvβ6-, but not by αvβ8-integrin requires the RGD motif in HSV gH

**Figure 8:**
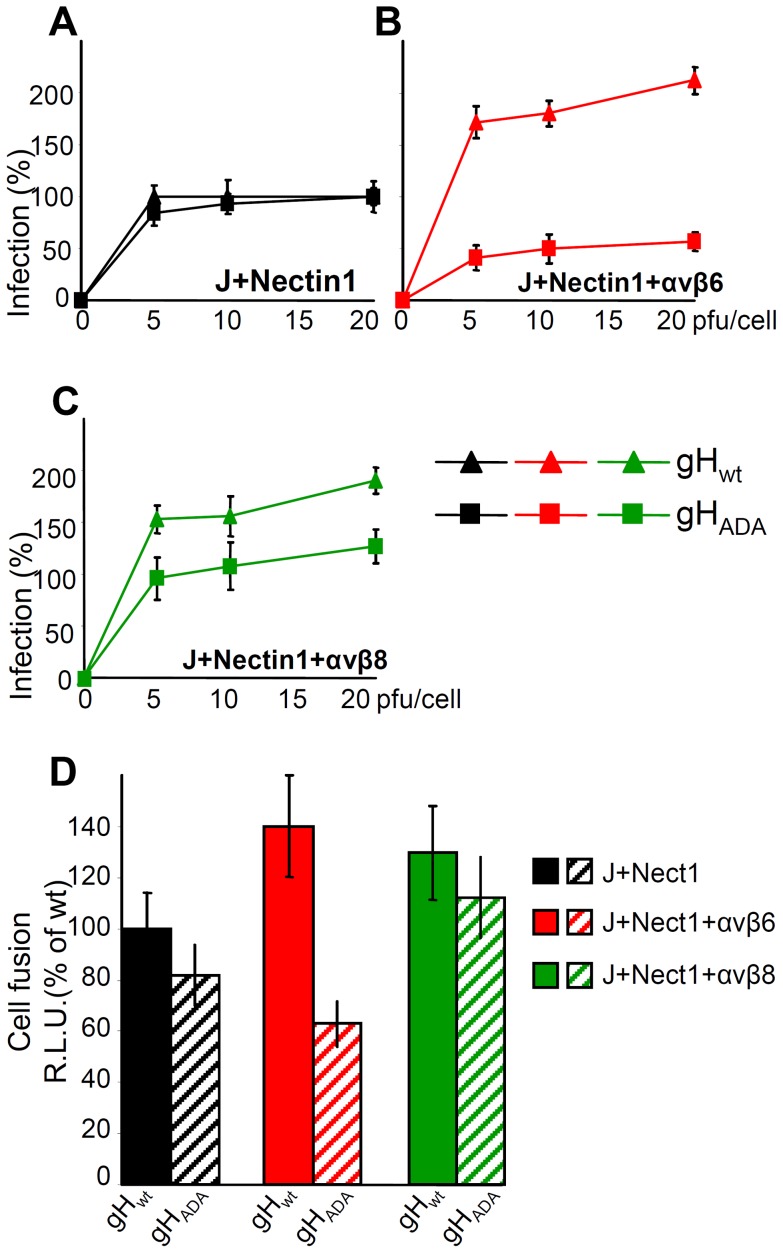
The RGD motif in gH is required for HSV infection and cell-cell fusion mediated by αvβ6–integrin, but not by αvβ8–integrin. (A–C). J cells were transfected with low amount of nectin1 alone (black triangle and square) (A), or nectin1 plus αvβ6-integrin (red triangle and square) (B), or nectin1 plus αvβ8-integrin (green triangle and square) (C), as detailed in legend to Fig. 7. 48 h later, cells were infected with increasing amounts (5–20 pfu/cell) of SCgHZ virus, a ΔgH HSV, complemented with gH_wt_ (black, red and green triangle) or with gH_ADA_ (black, red and green square), and harvested 16–18 h later. Extent of infection was expressed as detailed in legend to Fig. 3. Eeach point represents the average of triplicates. 100% of infection is the value obtained in J cells transfected with nectin1 alone and infected with 20 pfu of SCgHZ virus complemented with gH_wt_. Bars show SD. (D) Cell-to-cell fusion between effector J cells expressing gH_wt_, or gH_ADA_ plus the trio of gD, gL and gB, and luciferase, and target J cells expressing nectin1 alone, or nectin1 plus αvβ6-integrin, or nectin1 plus αvβ8-integrin plus Renilla luciferase. Fusion was quantified by means of a T7 promoter-driven reporter luciferase gene transfected in effector cells, and expressed as percentage relative luciferase units (R.L.U.). 100% is the value obtained in cells expressing nectin1 alone and gH_wt_ plus the trio of gD, gL, gB. Each point represents the average of triplicates. Bars show SD.

αv-integrins can interact with their ligands through a RGD motif. Because HSV gH carries an RDG motif at aa 176–178, we asked whether the interaction of αvβ6- or of αvβ8-integrin with gH occurs through this motif. A mutant form of gH in which the RGD motif was substituted to ADA [41] was used in two functional assays: infection and cell-to-cell fusion. For infection, the ΔgH HSV mutant ScgHZ [42] was grown in cell lines expressing gH_wt_ or gH_ADA_. The cell-expressed gH complements the deletion in the virus, and virions pseudotyped with gH_wt_ or gH_ADA_ are generated. J cells transfected with nectin1 plus αvβ6- or αvβ8-integrin, or with nectin1 alone, were infected with virions carrying gH_wt_ or gH_ADA_. Fig. 8 A–C shows that infection of J cells expressing nectin1 alone occurred irrespective of whether virions carried gH_wt_ or gH_ADA_, in agreement with a previous report [32]. In contrast, infection of J cells expressing nectin1 plus αvβ6-integrin was severely impaired when virions carried gH_ADA_. Infection of J cells expressing nectin1 plus αvβ8-integrin was only slightly inhibited when virions carried gH_ADA_.

The cell-to-cell fusion assay mimics virus-to-cell entry in that it requires the same four essential virion glycoproteins (gD, gH/gL, gB), and one of the gD receptors nectin1 or HVEM [43]. The effector cells are transfected with the quartet of gH/gL, gD, gB, plus T7-promoter-driven luciferase [44]. The target cells are transfected with the required glycoprotein receptor and T7-polymerase. In our assay the target J cells were transfected with gH_wt_ or gH_ADA_ plus the trio of gD, gL, gB. The effector cells were transfected with nectin1 alone, nectin1 plus αvβ6-integrin, or nectin1 plus αvβ8-integrin. Fig. 8 D shows that cell-to-cell fusion was increased by 40% or 30% when J cells expressed αvβ6-integrin or αvβ8-integrin, plus nectin1, relative to cells expressing nectin1 alone. This occurred with the wt allele of gH. When the gH_ADA_ substituted for gH_wt_ there was a dramatic inhibition in the αvβ6-integrin-enhanced fusion, but not in the αvβ8-integrin-enhanced fusion. The results of the two assays indicate that the gH interaction with αvβ6-, but not with the αvβ8-integrin, entails the RGD motif in gH.

### αvβ6-integrin and αvβ8-integrin enable specific endocytic pathways of HSV entry

HSV enters different cells by different pathways. αvβ3 integrin was identified in our laboratory as a cellular determinant in the choice of the pathway of entry, capable of routing HSV to a pathway dependent on lipid microdomains and dynamin 2, and proceeding to acidic endosomes [30]. Whether integrins other than αvβ3 are determinants in the choice of HSV entry pathways, and whether different integrins route HSV to different pathways is not known. To address these questions, we analyzed the effects of well known entry inhibitors on HSV infection of J cells expressing nectin1 alone, or nectin1 plus αvβ6-, αvβ8-, or αvβ3-integrins. J cells are suitable to address this question, since, when transfected with a gD receptor they enable a pathway of entry independent of lipid rafts and acidic endosome [45].

**Figure 9:**
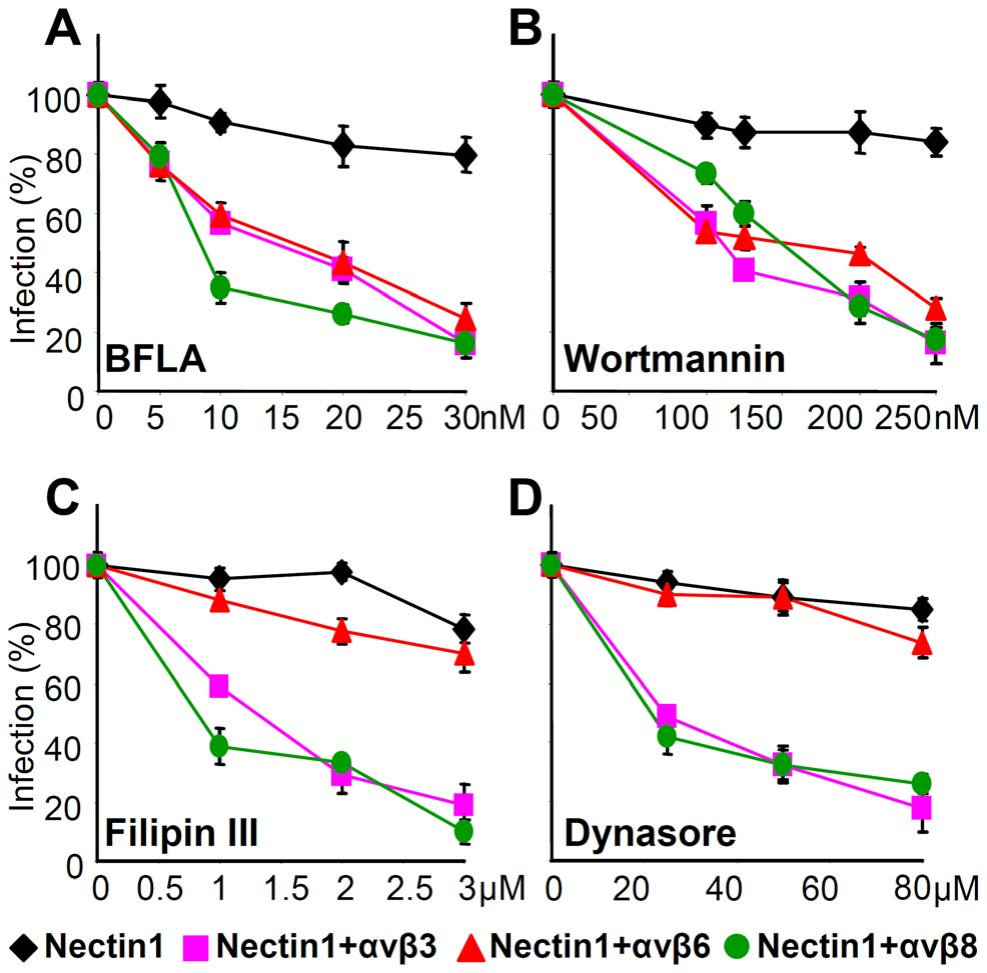
Effect of expression of αvβ6- or αvβ8-integrin on the pathway of HSV entry, defined through sensitivity to specific inhibitors. J cells were transfected with plasmids encoding nectin1 (black diamond), nectin1 plus αvβ3-integrin (pink square), nectin1 plus αvβ6-integrin (red triangle), or nectin1 plus αvβ8-integrin (green circle). 48 h after transfection cells were pretreated with increasing amounts of BFLA (A), wortmannin (B), filipin III (C) or dynasore (D) and infected with R8102 (3 pfu/cell) for 90 min at 37°C in the same medium. Inoculum was removed and cells were overlaid with Dulbecco's minimal essential medium (DMEM) for 6–8 h. Specific details were as follows. For filipin III and dynasore, cells were preincubated with the compounds at 37°C for 30 min or 60 min, respectively, and infected for 30 min (30 pfu/cell) in the same medium. Infected cells were overlaid without inhibitor. BFLA or wortmannin were present also after virus absorption. Infection was quantified as detailed in the legend to Fig. 3, and expressed as percentage of untreated cells. Bars show SD.

The significant inhibitors were bafilomycin A (BFLA), a specific inhibitor of the Na-H pump, hence of endosomal acidification, filipin III, an inhibitor of the lipid microdomain platforms, dynasore, an inhibitor of the dynamin 2 GTPase required to seal the endosomal invaginations and generate endosomes, and wortmannin, an inhibitor of phosphoinositide 3-kinase (PI3K). The results were as follows (Fig. 9). Infection of J cells expressing nectin1 alone was not sensitive to BFLA (Fig. 9 A), as reported [45], and occurs by fusion at plasma membrane or at a neutral pH compartment. Anyone of the three integrins - αvβ6, αvβ8 and αvβ3 – rendered the entry pathway sensitive to BFLA (Fig. 9 A). The wortmannin sensitivity closely mirrored that of BFLA, implying an involvement of PI3K when HSV entry occurs through acidic endosomes, but not at the plasma membrane or in a neutral compartment (Fig. 9 B). Filipin III did not inhibit infection in J cells expressing nectin1 alone (Fig. 9 C), but did inhibit infection in J cells expressing nectin1 plus αvβ8-, or αvβ3-integrin (Fig. 9 C). Remarkably, filipin III failed to inhibit infection of J cells expressing nectin1 plus αvβ6-integrin. The effect of dynasore closely overlapped that of filipin III (Fig. 9 D). The filipin III and dynasore sensitivity upon αvβ3-integrin expression was in agreement with our previous findings [30]. Cumulatively, the results indicate that both αvβ6- and αvβ8-integrin greatly impact on the HSV entry pathway. Both switch HSV to acidic endosome entry. αvβ8-, like αvβ3-integrin, routes HSV to a pathway dependent on lipid microdomain and dynamin2; in contrast, αvβ6-integrin routes HSV to a pathway independent of lipid microdomain and dynamin 2.

## Discussion

The epithelial αv group of integrins, also named as RGD receptors, includes αvβ3-, αvβ5-, αvβ6-, αvβ8-integrins, in addition to the αIIbβ3. We report that:

- αvβ6- and αvβ8-integrins play a critical role in HSV entry into cell lines which are models of epithelial, including keratynocytic, and neuronal cells. Each of the two integrins acts to promote HSV entry independently of the other. The two serve in an interchangeable manner. This conclusion rests on loss-of-function experiments, in which infection was inhibited by function-blocking MAbs or by integrin silencing through siRNAs, and on gain-of-function experiments, in which infection increased following expression of αvβ6 in integrin-negative K562 cells. The finding that the simultaneous silencing of αvβ6- and αvβ8-integrins reduced infection by about 90% emphasizes their critical role in HSV infection.
- Both αvβ6- and αvβ8-integrins bind gH/gL at high affinity. The binding is highly selective as αvβ5-integrin does not bind gH/gL at all. The binding sites of αvβ6- and of αvβ8-integrins in gH/gL differ; it is the RGD motif for αvβ6-integrin, but not for αvβ8-integrin. Consistent with the high affinity binding, both the αvβ6- and αvβ8-integrin contributed to attachment of virions to cells, a step which so far was ascribed to gC and gB binding to HS, and to gD binding to one its receptors. The contribution of αvβ6- or αvβ8-integrin to HSV attachment was much lower than that of heparan sulphate, whose absence reduces infection by about 100 fold [38] [39]. Together, the requirement for αvβ6- or αvβ8-integrin for infection and the high affinity binding are criteria to define αvβ6- and αvβ8-integrins as HSV receptors. Importantly, as shown also here, αvβ6- and αvβ8-integrins are preferentially expressed in epithelial cells, and β8-integrin is expressed at high levels in the central nervous system [34], [35]. Epithelial cells and central nervous system are major targets of HSV infection *in vivo*. Further yet, αvβ6 is up-regulated during tissue remodeling, including that accompanying wound healing [46], a condition that favors keratinocyte infection by HSV [47].
- The roles of αvβ6- or αvβ8-integrin as HSV receptors are in contrast to that of αvβ3-integrin. The latter binds HSV gH/gL at a 100 fold lower affinity [31], and serves as a routing factor, but not as a *bona fide* receptor; its silencing modified the route of infection but does not reduce it [31].
- αvβ6- or αvβ8-integrin did not substitute for the gD receptor nectin1. The role of integrins is therefore additional - and not alternative - to that of gD receptors. Altogether, HSV entry into cells requires both the interaction of gD with one of its receptors, and the gH/gL interaction with αvβ6- or αvβ8-integrin, a view which impacts on the current model of HSV entry, as detailed below
- The most dramatic effects exerted by αvβ6- and αvβ8-integrins were modifications of the pathway of HSV entry. Both integrins routed HSV from a plasma-membrane (or neutral pH compartment) portal of entry to acidic endosomes, and, concomitantly, to PI3K-dependency. Interestingly, the two integrins differed in the specific endocytic pathway they enabled. αvβ8-integrin enabled an entry pathway dependent on lipid microdomains and dynamin 2. αvβ6-integrin enabled a pathway of entry independent of lipid microdomains and dynamin 2.

Cumulatively, the novel features to emerge from this study are that HSV entry into epithelial cells requires the gH/gL interaction with one of the two interchangeable receptors, αvβ6-integrin or αvβ8-integrin. This interaction profoundly affects the pathway of entry, which takes the way of acidic endosomes. In the case of involvement of αvβ8-integrin, the portal of entry is located at lipid microdomains and necessitates dynamin 2. In the case of involvement of αvβ6-integrin, the portal of entry is located outside the lipid microdomains and does not necessitate dynamin 2.

**Figure 10:**
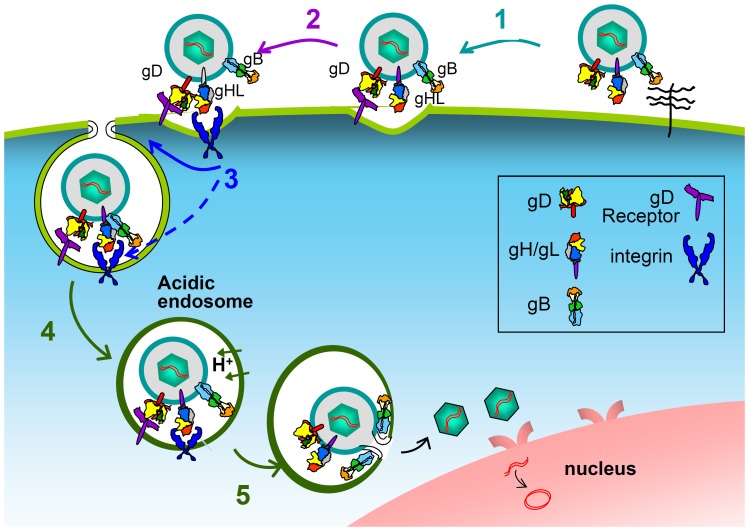
Schematic representation of the intervention of integrins in the process of HSV entry and in the cascade of activation of the fusion glycoproteins. Following HSV attachment to cells via heparan sulphate glycosaminoglycans (1), the interaction of gD with one of its receptors activates gD (2); the activation is propagated to gH/gL; the details of this interaction are still under investigation. Integrins intervene (3): they promote endocytosis, and, most likely, induce or contribute to conformational changes, hence to activation of gH/gL. The gH/gL activation is propagated to gB. Endosomal acidification (4). Fusion execution by gB (5). Color code of the glycoproteins as in [69]. gH/gL and gB are shown in the resolved post-fusion structures.

Two questions arise. *Why did HSV evolved to employ gH/gL receptors in addition to the gD receptors? And, why did HSV choose integrins as gH/gL receptors?* Two characteristics of the entry of HSV, and of herpesviruses in general, are the multipartite nature of the entry apparatus, and the possibility to enter cells through a variety of pathways. The currently accepted model on how the HSV multipartite entry/fusion apparatus mediates fusion of the virion envelope with the cell membranes envisions sequential activation steps in a cascade fashion [7], [8], [11], [12], [15]. Even though the details of the interactions are still being defined, there is consensus that, upon gD binding to one of its receptors, conformational changes to gD ensue [11], [12], such that gD interacts with, or anyway propagates the activation to gH/gL, and thereafter or simultaneously to gB [9], [10], [14]. Current results introduce the intervention of the integrin receptors in this process. We propose that, in addition to promoting endocytosis, the interaction of gH/gL with the integrin receptor contributes to induce conformational changes and activation of gH/gL, as seen in EBV [48] (Fig. 10). Consequently, integrins would act as a trigger point in the activation cascade of the HSV fusion glycoproteins. Indeed, by placing the step of gH/gL activation under the double control of receptor-activated gD and of the integrin trigger point, the virus would ensure that the premature activation of gB is prevented, and, importantly, that virion endocytosis is synchronized with the activation of the fusion apparatus. In particular, when entry occurs at the plasma membranes, the receptor-mediated gD activation propagates to gH/gL and gB, possibly in a very brief timescale, perhaps in a matter of fraction of seconds. In the case of HSV entry by endocytosis, there must be a pause in the activation cascade, or a checkpoint that prevents the premature activation of gH/gL and gB, before endocytosis has taken place. An activating receptor for gH – in this case integrins, which are machines specialized in promoting endocytosis [49] – is ideally suited to fulfill this synchronization (Fig. 10). The entry pathways exploited by HSV include low pH or neutral pH endosomes, as well as the plasma membrane, which is also a neutral pH compartment. As a consequence, HSV had to evolve a strategy for control of activation of the fusion glycoproteins independent of acidic pH. The integrin trigger point on gH/gL results in a control on fusion independent of acidic pH, and thus confers to the virus the flexibility to use alternative pathways in different cells, and expands the range of cells that HSV infects.

Up to now, among herpesviruses, integrins are known to serve as receptors for EBV and HCMV, which engage with integrins through gH/gL, and for HHV-8, which engages with integrins through gB [20]–[23]. In EBV, the integrin-gH/gL interaction triggers conformational changes to the glycoproteins, likely activating them [48]. Whether all human herpesviruses need integrins in some way to enter certain cell types is an attractive open possibility. All herpesviruses encode a multipartite fusion apparatus, and enter a number of cells by endocytosis. Importantly, for EBV, HCMV and HHV8, placing the activation of gH/gL (EBV, HCMV) or gB (HHV-8) under an integrin trigger point may well result in a synchronization of the final steps of activation of the glycoproteins responsible for fusion execution. While many viruses in other families make use of integrin receptors to promote endocytosis [50]–[52], most either lack an envelope, e.g. adenoviruses and reoviruses, or do not encode an entry system as complex as that of a herpesvirus. Their usage of integrins thus intrinsically differs from that of HSV, as entry is less dependent on checkpoints to control an activation cascade.

We recently found that αvβ3integrin - which functions as a routing factor and drives HSV entry to a pathway dependent on lipid microdomains, dynamin 2 and acidic endosomes [30], [29] - serves as a sensor of the virus. In cooperation with TLR2, it elicits a branch of the innate response that contributes to NF-κB activation and production of a specific set of cytokines, mainly IFNα and β [31]. The question naturally arises as to whether the engagement of αvβ6 or αvβ8-integrin as receptors initiates a similar inimical response to the virus. In turn, this raises the question of what selective advantage is provided to HSV by use of an integrin to control fusion. As noted in earlier studies, HSV has evolved to evade the innate response soon after the onset of viral protein synthesis; in particular, infected cell protein 0 (ICP0) and ICP27 provide the first line of evasion [53]–[56]. We conclude that, as highlighted in this work, HSV takes advantage of integrins to enable its endocytic entry and to exert a checkpoint on the cascade of glycoprotein activation. It subsequently counteracts the innate response, when virus entry is completed, by aid of the immediate early proteins.

## Materials and Methods

### Cells, viruses and soluble proteins

293T, HeLa, SK-N-SH and J (a derivative of BHK-TK^−^ cells lacking any HSV receptor) [5] cells were grown in Dulbecco's modified Eagle's medium (DMEM) containing 5% to 20% fetal bovine serum (FBS). Colon carcinoma SW480, HaCaT and K562 cells were grown in L15, DMEM 4.5% glucose and Iscove's modified Dulbecco's medium, respectively. The growth medium for K562_αvβ6_ (a gift from Dr S. Blystone) contained 750 µg/ml neomycin G418. F6 cells were a stably transformed Vero cell line expressing HSV-1 gH under the control of HSV-1 gD promoter [42]. R8102, a HSV-1 recombinant carrying LacZ under the control of the α27 promoter [5] and RLM5, a HSV-1(F) recombinant expressing green fluorescent protein (GFP) [57] were described. In the gH deletion mutant (ΔgH HSV) SCgHZ the gH gene was replaced with LacZ gene, the virus was grown and titrated in the complementing F6 cells [42]. 293-B6 AVAP [58] and 293-B8 AVAP cells [59] (a gift of Dr. Stephen Nishimura, University of California at San Francisco), which secrete, respectively, truncated αvβ6 and αvβ8 integrin conjugated to alkaline phosphatase (AP), were grown in DMEM (Sigma) supplemented with 10% fetal bovine serum and 1% nonessential amino acids. Insect Sf9 cells were grown in Sf900 II medium (Invitrogen) and infected at a multiplicity of 2 with equal ratios of baculoviruses expressing truncated forms of αv and β5 cloned in frame respectively with a fos or jun dimerization domain [60] (a gift of Dr. Glen Nemerow, Scripps Research Institute). Soluble truncated integrins were purified as previously described [48]. Soluble gH_t_/gL and gB_t_, carrying One-StrEP-Tag epitope for affinity chromatography purification, were described previously [33], [41]. One-StrEP-tagged green fluorescent protein (GFP_t_) was provided by IBA GmbH (Göttingen) and used as negative control.

### Plasmids

Plasmids encoding HSV-1 gD, gB, gL and gH all under the control of the cytomegalovirus (CMV) promoter were described [61]. gH_ADA_ carries the indicated substitutions in the RGD motif [41]. pCAGT7 contained the T7 RNA polymerase gene under the control of the CAG promoter and pT7EMCVLuc plasmid expressed the firefly luciferase under the control of the T7 promoter [62]. EGFR2NΔ (epithelial growth factor receptor 2 Δ), named Erb-2, carriers the extracellular domain and transmembrane sequence of rat HER-2/neu, and is deleted of the tyrosine kinase domain [63]. Plasmids encoding nectin1 [5], Renilla luciferase (Promega), αv-, β6- and β8-integrin were described [36], [64].

### Antibodies

MAb 2077Z to the αvβ6 integrin heterodimer is a function-blocking antibody from Chemicon. MAb 37E1 to αvβ8 integrin, MAb R1.302 to nectin1 and PAb to HVEM were gifts from Nushimura S.L [65], M. Lopez [5] and G. H. Cohen and R. Eisenberg [66]. MAb L230 is a function-blocking antibody directed to αv integrin [67]. For surface plasmon resonance analysis MAb LS-C44264 to placental alkaline phosphatase (LifeSpan BioScience) was used to capture integrins αvβ6AP, and αvβ8AP the non-blocking monoclonal antibody LM142 to αv integrin (Millipore) was used to capture integrin αvβ5.

### Surface plasmon resonance spectroscopy

The kinetic parameters of the interaction between gH/gL and integrins were measured with a Biacore 2000 instrument (Biacore AB). Antibodies were immobilized on a research-grade CM-5 sensor chip by amino coupling with 1,200 relative units (RU) as a target for immobilization and used to capture soluble integrins. The first flow cell (FC1) was always used as a reference (no antibody immobilized), and the signal from FC1 was automatically subtracted by Biacore software from data obtained from the other three flow cells. Measurements were made at 25°C. Integrins were injected at a flow rate of 10 to 20 µl/min; gH/gL was injected immediately after integrin capture by using the low-dispersal injection setting (kinject) at a flow rate of 50 µl/min. After each run, the sensor chip surface was regenerated by injection of 6 M guanidine chloride in 25 mM HEPES-NaOH, pH 7.2 (50 µl at a flow rate of 100 µl/min), followed by additional washing. The baseline remained stable during 30 to 40 runs. Integrins and gH/gL were centrifuged before use and diluted in running buffer if necessary (10 mM HEPES-NaOH, pH 7.4, 150 mM NaCl, 0.005% surfactant P20 (Biacore, GE Healthcare). Each sample was degassed before injection.

### Curve fitting

BIAevaluation 4.1 software was used for the Biacore trace alignments and to zero the baseline. All traces were in full accordance with a 1∶1 Langmuir model. Formation and dissociation sections of Biacore traces fit a single-exponential function which was accepted as a model. Least-squares fitting of data and determinations of standard errors of the fitted parameters were conducted using the program KaleidaGraph (Synergy Software, Reading, PA).

### β6- and β8-integrin silencing

One target plus siRNAβ6 or β8 (Dharmacon, ON-TARGET plus, smart pool) were trasfected into 293T, HeLA or SW480 cells, 50 nM for each siRNA, by means of Dharmafect I, according to Dharmacon protocol for adherent cells. Control-silenced cells were transfected with siRNA to E.coli-polA_0054 (CGC GUG AUA UGC GAC GCG AUA AAG) synthesized by IBA Gmbh.

### qRT-PCR

Total RNA was purified by means of Total RNA Isolation kit (Macherey-Nagel) and reverse transcribed with high-capacity cDNA reverse transcription kit (Applied Biosystem). Real-time PCR primers were the inventoried TaqMan gene expression assay ITGB6 (Hs00168458_m1), ITGB8 (Hs00174456_m1) ITGAV (Hs00233808_m1) (Applied Biosystem). Quantitative Real Time PCR (q-RT-PCR) was performed as described [31].

### Inhibition of HSV infection by MAbs to αvβ6 or αvβ8-integrins and in integrin-silenced cells

293T, HeLa, SW480, HaCaT and SK-N-SH cells in 96 wells were preincubated with increasing amounts of MAb R1.302 to nectin1, MAb 2077Z to αvβ6-integrin, MAb 37E1 to αvβ8-integrin, or with mouse IgGs for 60 min at 37°C. R8102 (3 pfu/cell) was added to the MAb-containg medium for additional 90 min. Viral inoculum was removed, and cells were overlaid with DMEM containing MAbs for 6–8 h. Control-silenced, β6-integrin-silenced or β8-integrin-silenced 293T, HeLA or SW480 cells were infected with R8102 (3 pfu/cell) for 90 min at 37°C. The viral inoculum was removed and cells were overlaid with DMEM 1% FBS for 6–8 h. The extent of infection was assessed through β-galactosidase (β-Gal) activity at 405 nm, by means of o-nitrophenyl-β-D-galactopyranoside (ONPG), or by in situ staining with 5-bromo-4-chloro-3-indolyl-β-D-galactopyranoside [5]. K562_αvβ6_ were preincubated or not with 40 ug/ml of MAb 2077Z to αvβ6-integrin, MAb L230 to αv-integrin, or PAb R140 to HVEM for 1 h at 37°C. K562 and K562_αvβ6_ cells were infected with RLM5 (20 pfu/cell) in 3 ml for 90 min. The viral inoculum was removed and cells were overlaid with DMEM containing or not the indicated antibodies for 6 h. Control-silenced, β6-integrin-silenced, β8-integrin-silenced or double-silenced 293T cells were infected with RLM5 (3 pfu/cells) for 90 min at 37°C. Viral inoculum was removed; the extent of infection was assessed through EGFP expression in infected cells, 6 h after infection.

### Flow cytometry

Expression of αvβ6 or αvβ8–integrins in 293T, HeLA and SW480 cells was determined by FACS. In 293T cells (control-silenced and integrin-silenced) integrin expression was determined 72 h after transfection with siRNActrl, siRNAβ6, siRNAβ8 or a mixture of both. Cells were washed once with PBS (phosphate buffered saline) and once with PBS containing 10% FBS and allowed to react with MAb 2077Z to αvβ6-integrin, MAb 37E1 to αvβ8-integrin heterodimers for 1 h at 4°C. Cells were washed three times with PBS and allowed to react with phycoerythrin conjugated secondary antibody (Becton-Dickinson Pharmingen). Where indicated, cells were infected with RLM5, and infection was quantified through EGFP expression at 6 h after infection. The inhibition of HSV infection by MAb 2077Z to αvβ6-integrin, MAb L230 to αv-integrin and PAb to HVEM in K562 and K562_αvβ6_ cells was quantified through enhanced green fluorescent protein (EGFP) expression from the RLM5. Cytofluorimetric analysis was performed using a FACScalibur cytometer (BD), equipped with an argon laser on a minimum of 10,000 cells per sample, acquired in list mode.

### Inhibition of infection by pharmacological inhibitors

The stock solutions of filipin III (2.5 mM), dynasore (100 mM), bafilomycin A (BFLA) (160 mM) and wortmannin (2 M) (all from Sigma Aldrich) in dimethyl sulfoxide were stored at -20°C. Cells were exposed to the inhibitors for 1 h at 37°C and then infected with R8102 (3 pfu/cell) for 90 min in the presence of inhibitors. The viral inoculum was removed, and the cells were overlaid with medium containing inhibitors for 6–8 h. For filipin III and dynasore, cells were preincubated with the compounds at 37°C for 30 min or 60 min, respectively, and infected for 30 min (30 pfu/cell) in the same medium. Viral inoculum was removed; infected cells were overlaid without inhibitor and harvested 6–8 h after infection.

### Virions complemented with gH_wt_ or gH_ADA_

293T cells in T_150_ flasks were transfected with gH_wt_ or gH_ADA_ plus gL. After 4 h, cells were infected with a gH−/+ stock of SCgHZ (5 pfu/cell) [42]. Cell-absorbed virions were inactivated by a 1 min rinse with 40 mM sodium citrate-10 mM KCl-135 mM NaCl (pH 3). The monolayers were then rinsed twice with PBS and overlaid with medium containing 1% fetal calf serum. Cells were incubated overnight at 37°C. Extracellular virions were harvested, pelletted by high speed centrifugation, and titrated in F6 cells.

### Contribution of αvβ6- or αvβ8-integrin to HSV infection

J cells were transfected with plasmids encoding nectin1 (300 ng DNA/24 well), αv plus β6, or αv plus β8 integrins (300 ng each/24 well) by means of Lipofect 2000 (Life Technologies). Alternatively, J cells were transfected with low amount of nectin1 plasmid (75 ng DNA/24 well), plus or minus αv+β6 or αv+β8-integrins (300 ng DNA/24 well). The total amount of transfected plasmid DNA was made equal (675 ng/24 well) by the addition of Erb-2 plasmid DNA. 48 h after transfection, cells were infected with increasing MOI (2.5–30 pfu/cell) of R8102, or of SCgHZ virions complemented with gH_wt_ or with gH_ADA_, for 90 min at 37°C. After infection cells were overlaid with DMEM and harvested 16–18 h after infection.

### Cell-cell fusion assay

The luciferase-based cell-cell fusion assay was performed as described [44], with small modifications. Effector J cells were transfected with plasmid encoding gL, gD, gB, gH_wt_ or gH_ADA_. Target J cells were transfected with Renilla luciferase, low amount of nectin1 (75 ng DNA/24 well), plus or minus αv+β6, or αv+β8 (300 ng DNA for each plasmid/24 well). The total amount of plasmid DNA transfected was made equal by the addition of Erb-2 plasmid DNA. 24 h after transfection, effector and target cells were co-cultured for additional 24 h. Fusion was quantified by means of the T7 promoter-driven reporter luciferase gene; the amount of lysate was quantified by Renilla luciferase. Quantification was performed by means of Dual luciferase report assay (Promega). The extent of fusion was expressed as relative luciferase units (R.L.U.); 100% represents the extent of fusion induced by gH_wt_ in cells expressing nectin1 alone.

### Binding of to cell surface by CELISA

Cells grown in 96-well were incubated for 1 h at 4°C with One-StrEP tagged gB_t_, gH_t_/gL, or GFP_t_ (2 µM) in DMEM containing 5% FBS and 30 mM HEPES [68], washed three times with the same buffer, and further incubated for 1 h at 4°C with HRP (horse radish peroxidase)-conjugated MAb to One-StrEP tag (*Strep*-Tactin) (IBA GmbH, Göttingen). Following three additional rinsings, cells were reacted with *o*-phenylenediamine (Sigma-Aldrich) at 0.5 mg/ml; the optical density was read at 490 nm. For heparin inhibition studies glycoproteins were preincubated for 1 h at 4°C with 5 µg/ml heparin (Sigma-Aldrich), prior to addition to cells.
